# Far-UVC (222 nm) efficiently inactivates an airborne pathogen in a room-sized chamber

**DOI:** 10.1038/s41598-022-08462-z

**Published:** 2022-03-23

**Authors:** Ewan Eadie, Waseem Hiwar, Louise Fletcher, Emma Tidswell, Paul O’Mahoney, Manuela Buonanno, David Welch, Catherine S. Adamson, David J. Brenner, Catherine Noakes, Kenneth Wood

**Affiliations:** 1 NHS Tayside, Photobiology Unit, Ninewells Hospital and Medical School, Dundee, DD1 9SY UK; 2 School of Civil Engineering, University of Leeds, Leeds, LS2 9JT UK; 3 School of Medicine Ninewells Hospital and Medical School, University of Dundee, Dundee, DD1 9SY UK; 4 Center for Radiological Research, Columbia University Medical Center, New York, NY USA; 5 School of Biology, Biomedical Sciences Research Complex, University of St Andrews, North Haugh, St Andrews, KY16 9ST UK; 6 SUPA, School of Physics and Astronomy, University of St Andrews, North Haugh, St Andrews, KY16 9SS UK

**Keywords:** Public health, Influenza virus, Tuberculosis, Viral infection, Antimicrobials, Bacteria, Air microbiology, Pathogens, Civil engineering, Lasers, LEDs and light sources

## Abstract

Many infectious diseases, including COVID-19, are transmitted by airborne pathogens. There is a need for effective environmental control measures which, ideally, are not reliant on human behaviour. One potential solution is Krypton Chloride (KrCl) excimer lamps (often referred to as Far-UVC), which can efficiently inactivate pathogens, such as coronaviruses and influenza, in air. Research demonstrates that when KrCl lamps are filtered to remove longer-wavelength ultraviolet emissions they do not induce acute reactions in the skin or eyes, nor delayed effects such as skin cancer. While there is laboratory evidence for Far-UVC efficacy, there is limited evidence in full-sized rooms. For the first time, we show that Far-UVC deployed in a room-sized chamber effectively inactivates aerosolised *Staphylococcus aureus*. At a room ventilation rate of 3 air-changes-per-hour (ACH), with 5 filtered-sources the steady-state pathogen load was reduced by 98.4% providing an additional 184 equivalent air changes (eACH). This reduction was achieved using Far-UVC irradiances consistent with current American Conference of Governmental Industrial Hygienists threshold limit values for skin for a continuous 8-h exposure. Our data indicate that Far-UVC is likely to be more effective against common airborne viruses, including SARS-CoV-2, than bacteria and should thus be an effective and “hands-off” technology to reduce airborne disease transmission. The findings provide room-scale data to support the design and development of effective Far-UVC systems.

## Introduction

Severe acute respiratory coronavirus 2 (SARS-CoV-2), the virus responsible for the COVID-19 pandemic, can be transmitted by a single individual to one or more people through viral transport in airborne particles^1–4^. The risk of airborne SARS-CoV-2 transmission, from such events, increases in indoor environments where large groups of people congregate, especially when the environment is poorly ventilated^5,6^.

As has been well documented, the high levels of SARS-CoV-2 transmission have overwhelmed national healthcare systems, resulted in millions of deaths and caused long term health problems. The impact on the global economy has been, and will continue to be, devastating, which in turn has resulted in further welfare and public health issues.

It is therefore clear that reducing or preventing SARS-CoV-2 transmission is a critical and unprecedented global challenge. Transmission control measures have included national lockdowns, restrictions on social and business gatherings, improved indoor ventilation, public health campaigns, protective face coverings and vaccination. These control measures have different success rates and each comes with its own challenges. Vaccination has been one of the most effective measures in reducing death and serious illness, although the evidence is unclear on the efficacy of vaccination in reducing disease transmission^7,8^. Face coverings can be an effective control measure for reducing the risk of airborne transmission but rely on individual behavioural choices, with high levels of compliance required to achieve population level impacts on transmission^9,10^. As the COVID-19 pandemic progresses in time, there is lower acceptance and adoption of control measures that impact on daily life, and therefore an increased need for effective measures that do not rely upon human behavioural choices^11,12^. This is also important beyond COVID-19; airborne transmission has been recognised as an important mechanism for a wide range of other viral infections including influenza, measles, other human coronaviruses (SARS-CoV, Middle East Respiratory Syndrome MERS-CoV) and Respiratory Syncytial Virus (RSV) as well as for bacterial infections including Tuberculosis and some pathogens responsible for hospital acquired infections^13–15^.

Germicidal ultraviolet (GUV) is a control measure which meets the above requirements, with a scientific track record of success. In 1942, Wells et al.^16^ demonstrated less transmission of measles and mumps between children within upper-room GUV irradiated classrooms compared to control groups in rooms without GUV. Similarly, Escombe et al.^15^ demonstrated a greater than 70% reduction in transmission of tuberculosis from patients to guinea pigs when upper-room GUV was utilised, with 35% tuberculosis infection in control group and 9.5% infection in the group with GUV. However a major challenge for conventional 254 nm GUV is accidental exposure of humans, which can result in potentially painful sunburn-type reactions in the skin and cornea^17^. This limits traditional GUV to carefully designed upper-room systems, enclosed units or to irradiation of unoccupied rooms^18^. Even when adopted in this manner, accidental exposures can still occur and affect technology adoption^19,20^.

A potential solution is ‘Far-UVC’, germicidal ultraviolet-C radiation typically in the wavelength range from 200 to 230 nm. A common source of Far-UVC is Krypton Chloride (KrCl) excimer lamps with a primary emission wavelength of 222 nm, and low residual emission throughout the ultraviolet region of the electromagnetic spectrum^21^. The germicidal properties of KrCl excimer lamps have been shown in laboratory experiments to inactivate gram-positive and gram-negative bacteria, drug-resistant bacteria, influenza viruses and human coronaviruses including the SARS-CoV-2 virus^22–28^. Importantly, when filtered to minimise ultraviolet emissions at wavelengths longer than 230 nm, KrCl Far-UVC excimer lamps are much less likely than conventional (254 nm) GUV sources to induce acute adverse reactions on skin and eyes, and studies to date in animal and human models have not demonstrated any long-term adverse health effects^21,29–34^.

Whilst the laboratory results are encouraging, inactivation of a pathogen in a controlled bench-scale laboratory environment does not necessarily translate into reduced disease transmission when the technology is deployed with ‘real-world’ limitations^35,36^. Historical precedent with upper-room GUV provides some confidence in the potential for Far-UVC to reduce disease transmission, however there remains an unmet need for real-world evaluations^16,37^. Such studies are complex and must be performed over prolonged periods of time (typically at least 12 months). A translational step towards real-world studies are experiments in large, room-sized, aerosol chambers. These room-sized chambers, with controlled air-flow, temperature and humidity are designed to replicate a real-room environment. Such spaces have been used to demonstrate the effectiveness of upper-room GUV systems and to study the survival and dispersion of microorganisms^38–43^. They can also provide significant insight into the application of technologies in rooms where an infectious person may be present over a prolonged period of time, a situation that is common in schools, workplaces, hospitals and hospitality venues. With the continual controlled release of airborne pathogen, achieving a steady-state environment, the air within the chamber can be regularly sampled both with and without the environmental air disinfection technologies, providing an indication closer to real-world performance. Here we investigate for the first time the efficacy of Far-UVC for inactivating an airborne pathogen under steady-state conditions in a full-scale room-sized bioaerosol chamber.

## Results

**Figure 1:**
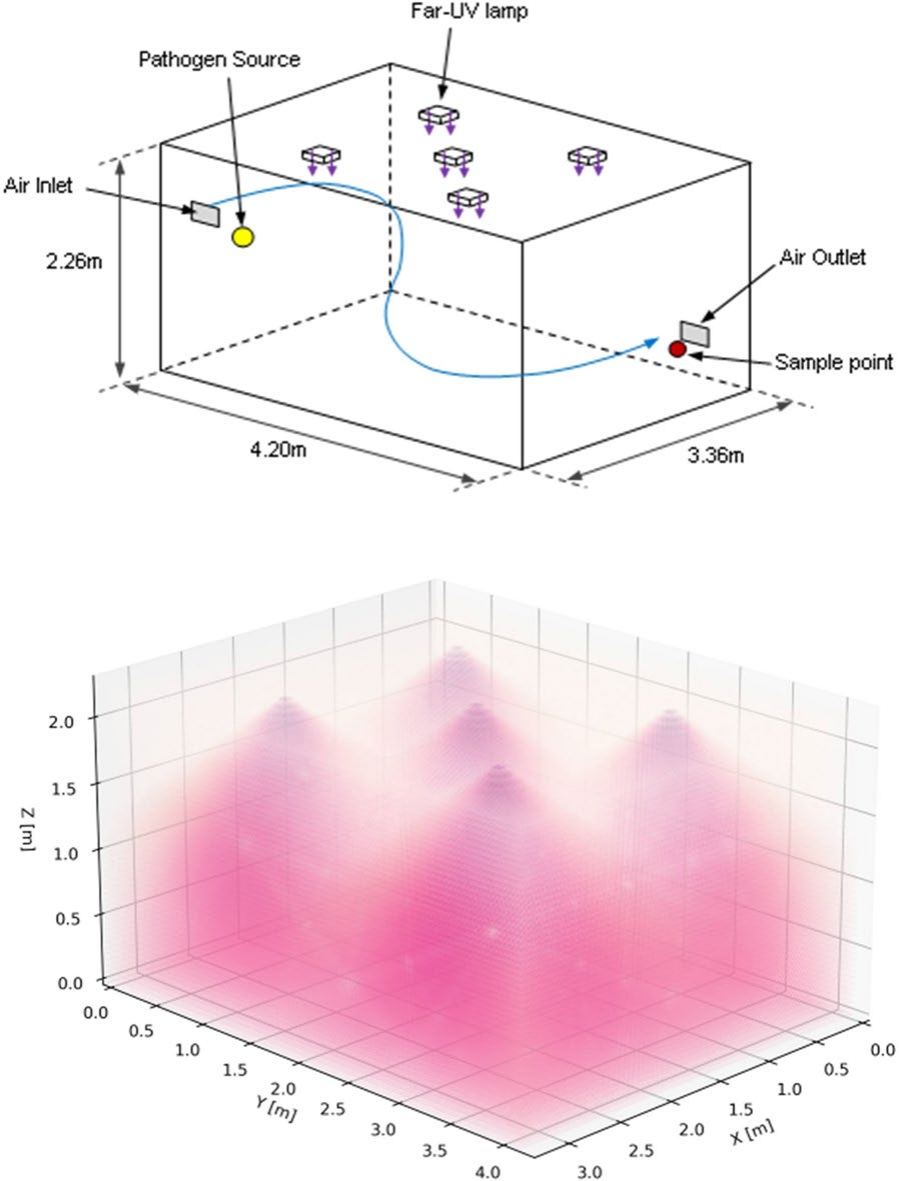
3D schematics of the bioaerosol chamber configuration showing room dimensions, the position of the lamps, pathogen source and collection point (top) with an illustrative example of the Far-UVC lamp emissions (bottom).

Five Krypton Chloride excimer lamps, filtered to reduce ultraviolet emissions at wavelengths longer than 230 nm and with diffusers secured at their emission window to broaden their irradiation pattern, were secured to the ceiling of a room-sized bioaerosol chamber at the University of Leeds.The lamps were arranged in a quincunx pattern (Fig. 1) with their emission directed towards the floor. Studies were undertaken either with all five lamps on or with only the central lamp on. This was done to investigate the effect of partial (one lamp) and full (five lamp) irradiation of the room volume. The mechanically ventilated 32 m^3^ chamber was operated at a ventilation rate of three air-changes-per-hour (ACH) and a continuous release of aerosolised *Staphylococcus aureus* was introduced to the room at a height of 168 cm. After a 60 min stabilisation period, 10 air samples were taken over a 50 min period. Then either one (the central Far-UVC lamp) or five Far-UVC sources were switched on and the sampling continued for a further 50 min.

These measurements were repeated using 3 different lamp exposure rates (Table 1). The exposure rates chosen were motivated by existing International Commission on Non-Ionising Radiation Protection (ICNIRP) guidelines for exposure to optical radiation (“Medium” scenario) and American Conference of Governmental Industrial Hygienists (ACGIH) threshold limit values (“High” scenario)^17,44^. An additional scenario at much lower lamp intensity was also included (“Low” scenario). Statistical analysis is detailed in Table S1.

**Table 1:** Average percentage pathogen reduction, irradiance and calculated 8-h exposure dose for three different exposure conditions at two heights from the ground.

		Peak values				Average values				Average % pathogen reduction (SD)
		Height = 1.7 m		Height = 1 m		Height = 1.7 m		Height = 1 m		
		Irradiance (µWcm^-2^)	8-h dose (mJcm^-2^)	Irradiance (µWcm^-2^)	8-h dose (mJcm^-2^)	Irradiance (µWcm^-2^)	8-h dose (mJcm^-2^)	Irradiance (µWcm^-2^)	8-h dose (mJcm^-2^)	
High	1 lamp	14.4	***415***	1.93	***56***	0.57	16.5	0.45	12.9	93.7**** (1.0)
	5 lamps	14.4	***415***	3.42	***98***	2.73	***78***	2.01	***58***	98.4**** (0.7)
Medium	1 lamp	0.92	***26.5***	0.13	3.7	0.03	0.87	0.03	0.82	65.9**** (4.0)
	5 lamps	0.92	***26.5***	0.22	6.3	0.14	4.1	0.13	3.67	92.0**** (0.9)
Low	1 lamp	0.09	2.65	0.01	0.37	0.003	0.09	0.003	0.08	12.8^ ns^ (3.8)
	5 lamps	0.09	2.65	0.02	0.63	0.01	0.41	0.01	0.37	28.7** (3.4)

The bold, italicised 8-h exposure values are above the ICNIRP 222-nm exposure limit of 23 mJcm^-2^. No exposures exceeded the 2022 ACGIH threshold limit value for skin of 478 mJcm^-2^ at 222 nm. Statistical significance is represented by: ns = p > 0.05, * = p ≤ 0.05, ** = p ≤ 0.01, *** = p ≤ 0.001, and **** = p ≤ 0.0001.

**Figure 2:**
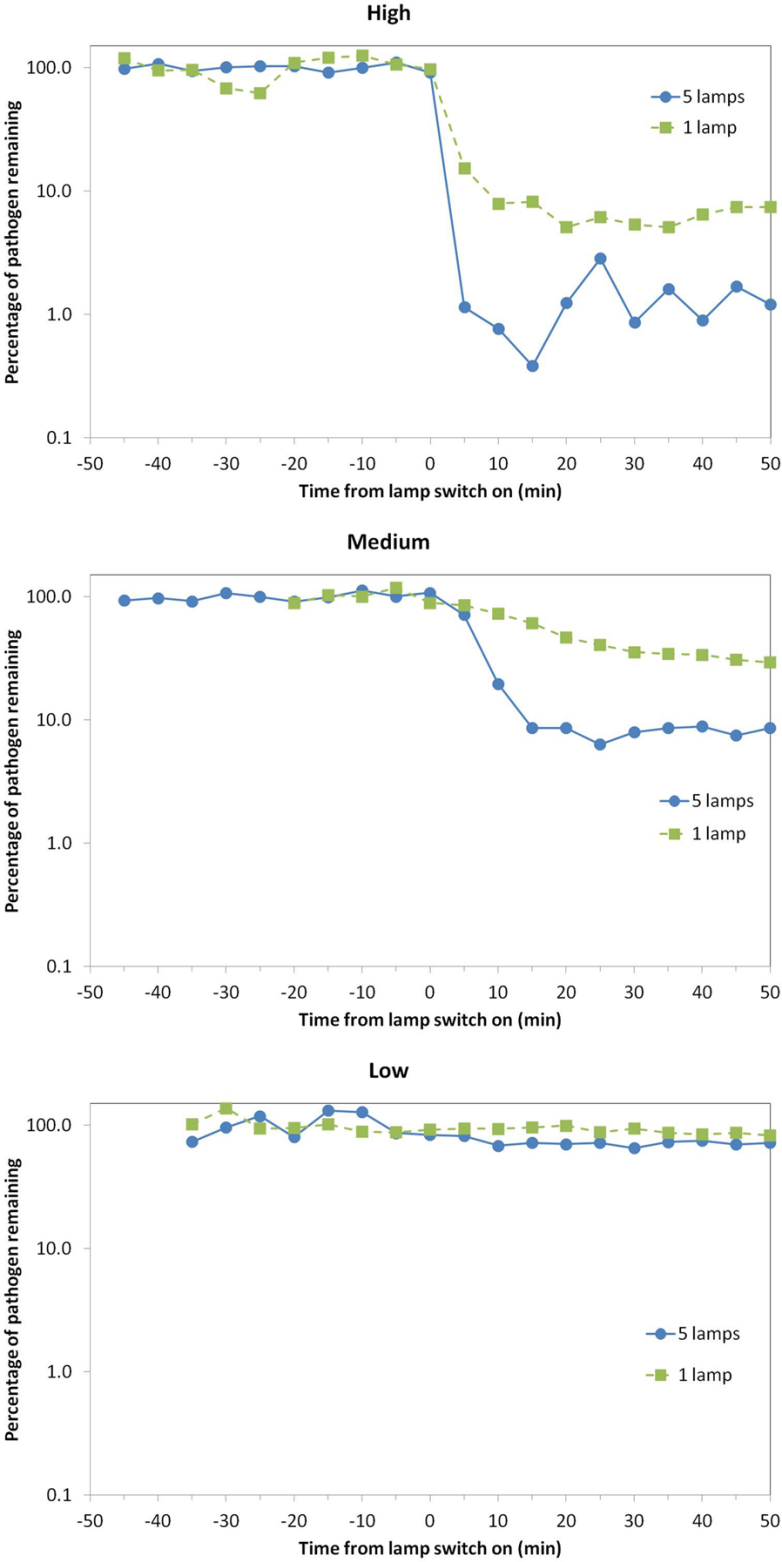
Percentage of viable airborne S. aureus remaining plotted on a logarithmic y-axis against time after switch-on of the Far-UVC sources for three different exposure scenarios—high (top), medium (middle) and low (bottom). Note that the pathogen was continuously released into the room throughout the experiment: The studies were undertaken using either a single central lamp (green, square data points, dashed lines) or all five Far-UVC lamps (blue, circular data points, solid lines).

As described in the “Methods” sections, the concentration of viable *S. aureus* pathogens in air at the collection location (Fig. 1), was serially sampled for 4 min every 5 min, both before and after the lamps were switched on (“lamp on”). The results, quantified as colony forming units per cubic metre (cfu m^-3^), are shown in Fig. 2 and Table 1, both for the 45 min prior to ”lamp on”, and serially for 50 min after “lamp on”. The values after “lamp on” are expressed as percentages of the average values prior to lamp on. Again it is emphasized that the pathogen was continuously released into the room throughout the experiment.

As expected, the highest reduction in the steady-state airborne viable *S. aureus* load was with the “High” exposure scenario. Using all five lamps this reduced the steady-state viable pathogen load by 98.4% (standard deviation 0.7%) compared to ventilation alone (three air-changes-per-hour). This produced an estimated equivalent air change rate range of 128–322 eACH (one standard deviation confidence interval). The peak 8-h exposure dose in this “High” scenario is motivated by, the American Conference of Governmental Industrial Hygienists (ACGIH) Threshold Limit Value (TLV) for the skin (478 mJcm^-2^ at 222 nm over 8 h)^42^. A single lamp in the “High” exposure scenario did not exceed the ICNIRP exposure limit for an average 8-h exposure dose and reduced pathogen load by 93.7%, which produces an estimated equivalent air change rate range of 33–66 eACH. Although the single lamp did not irradiate the full room, good air mixing in the chamber is likely to have resulted in this very substantial effect.

The “Medium” exposure scenario, with a peak 8-h exposure dose motivated by the current ICNIRP guideline exposure limit at 222 nm of 23 mJcm^-2^, produced a 92.0% reduction in the steady state viable pathogen load using all five lamps. This corresponds to a mean 35 eACH (range 27–46 eACH), equivalent to over 11 times the baseline ventilation with new steady state reached in under 15 min. It is relevant to note that while the 8-h *peak* exposure dose is slightly higher than the ICNIRP guidelines exposure limits, the *average* 8-h exposure dose was more than 5 times lower (Table 1).

The “Low” exposure scenario, with very low intensity Far-UVC exposure rates (a factor of 10 lower than the “Medium” exposure rate scenario), produced a 13% (one lamp) and 29% (five lamps) reduction in viable pathogen load.

## Discussion

**Figure 3:**
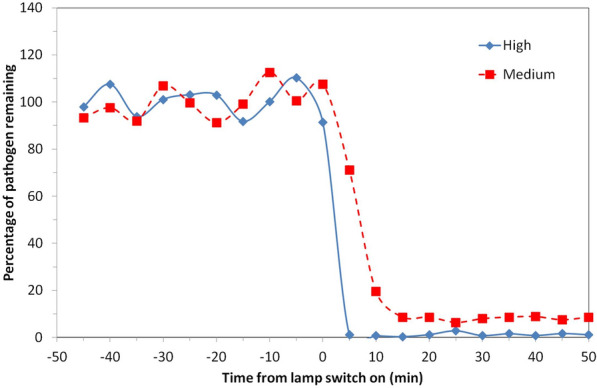
Percentage of viable airborne S. aureus remaining plotted on a linear y-axis for two of the exposure scenarios motivated by ICNIRP guideline exposure limits (5 lamps “Medium”) and ACGIH Threshold Limit Values (5 lamps “High”). Note that the pathogen was continuously released into the room throughout the experiment with a mechanical ventilation rate of 3 air changes per hour.

We have demonstrated for the first time in a realistically sized room, with typical ventilation and a continuous source of airborne pathogens, the potential for Far-UVC to rapidly produce significant reductions in airborne pathogens. With the lamp intensities at a level where the ICNIRP guideline exposure limits would not be exceeded, a ~ 92% reduction in viable pathogens was demonstrated, taking less than 15 min to reach the new ambient level. At the ACGIH threshold limit values a ~ 98% reduction was demonstrated, taking less than 5 min. A comparison of the two scenarios described is shown in Fig. 3.

**Figure 4:**
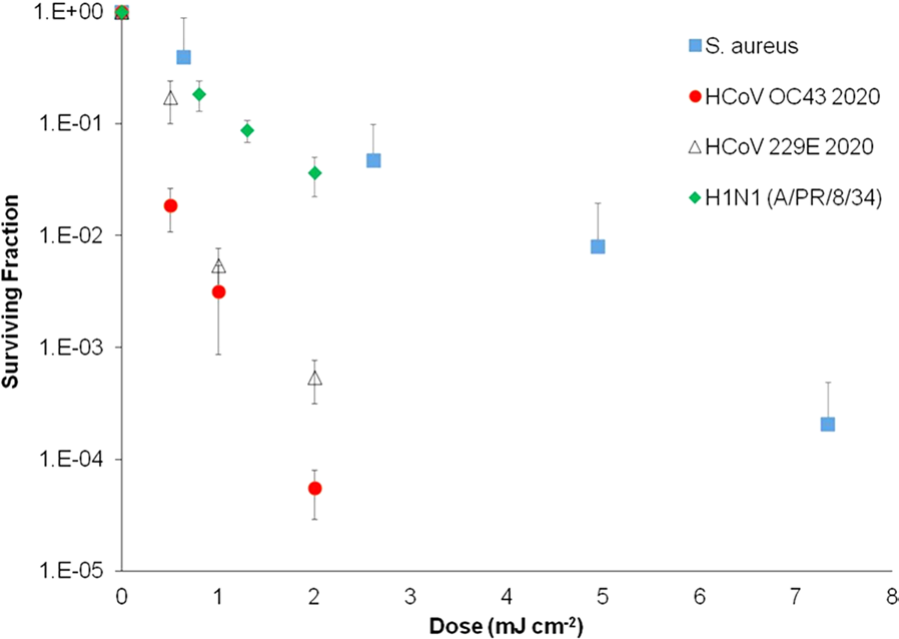
Inactivation of aerosolized human coronaviruses HCoV OC43 and HCoV 229E and H1N1 influenza virus at relevant low far-UVC doses, compared with aerosolized S. aureus with a Far-UVC lamp. Measurement taken at the Columbia University laboratory-based aerosolized pathogen irradiation system. HCoV OC43, HCoV 229E and H1N1 influenza data were published previously and included for comparison.

Although our study was not performed with SARS-CoV-2 for safety reasons, aerosolised *S. aureus* pathogen was used as a surrogate for more relevant (in the current context) airborne viruses such as human coronaviruses and influenza viruses. The rationale for this is shown in Fig. 4, where inactivation rates by Far-UVC of airborne human coronaviruses (OC43 and 229E), airborne influenza virus (H1N1), and airborne *S. aureus* are compared^27,28^. All these inactivation rates were measured using the same laboratory setup. No corresponding results have been reported for Far-UVC inactivation of airborne SARS-CoV-2, but corresponding results for Far-UVC inactivation of SARS-CoV-2 on surfaces suggest similar sensitivity to human coronaviruses OC43 and 229E^45–47^. Our results demonstrate that airborne *S. aureus* is less sensitive to inactivation by Far-UVC than airborne influenza and human coronaviruses, from which we conclude that *S. aureus* is a conservative surrogate. It is hypothesised that percentage reductions achievable for airborne coronavirus and airborne influenza virus would likely be larger, and have shorter inactivation times.

For installers of Far-UVC it may be challenging to interpret and apply optical radiation exposure limits^17,44^. Many will opt for the conservative approach of assuming an 8-h exposure at the peak irradiance. However exposure limits are intended to be used with a Time Weighted Average (TWA) irradiance (E_TWA_), which considers the actual exposure an individual has received within a space^48^. This allows for a higher peak irradiance if the E_TWA_ remains within limits. In this study, the peak lamp intensities could have been five times higher than the “Medium” scenario, thereby improving inactivation, and the average 8-h dose would still be within ICNIRP guideline exposure limits.

This highlights the importance of correct installation of Far-UVC, to ensure the designated space is appropriately and safely irradiated. For example, whilst a single lamp in the “High” scenario produced an overall ~ 94% pathogen reduction, there were areas of the chamber which were not fully irradiated. For real rooms, which may be larger and have potentially less effective air mixing than the chamber used in these experiments, the actual pathogen reduction may be significantly lower. As a result of previous modelling studies, we modified the Far-UVC lamps by placing a diffusing material at the emission surface of the Far-UVC sources within the chamber to broaden their irradiation pattern and increase Far-UVC coverage^49^.

Our results also provide some initial data that enable comparison to other technologies such as portable air cleaners. These typically have a clean air delivery rate (CADR) between 200 and 500 m^3^h^-1^ depending on the size of units. For the experimental chamber this would result in between 6.2 and 15.5 eACH. Therefore the “Medium” Far-UVC scenario with 5 lamps performs substantially better than even a higher flow HEPA based air cleaner. Although the design and installation of a Far-UVC system has a higher degree of complexity than a “plug and play” portable air cleaner, the approach has the potential to offer far greater eACH and is also silent. Another potential advantage of Far-UVC over air cleaners and upper-room GUV is that it may not require “good” air mixing within the room. We will be investigating this in future research.

All methodologies designed to reduce airborne transmission of diseases such as COVID-19 would ideally be used within a layered approach involving, as appropriate, vaccination, social distancing, masks and ventilation. Further work is required to explore the influence of parameters such as temperature, humidity, ventilation rates and proximity to infectious source but the results reported here should provide confidence that Far-UVC, when deployed appropriately, and conforming to current (or future) safety regulatory limits, is likely to be an effective, human behaviour independent, control measure to inactivate key airborne pathogens such as human coronavirus and influenza—and thus reduce the airborne, and potentially surface, transmission of these diseases.

## Methods

### Bioaerosol chamber

Experiments were conducted in a controlled bioaerosol chamber with dimensions 4.26 m in length, 3.35 m width and a height of 2.26 m. The chamber is mechanically ventilated and operated under negative pressure with a full fresh air system that is HEPA filtered on the supply and extract to provide both experimental control and safety in operation. Ventilation air was supplied through a high level wall mounted inlet grille located in one corner of the room. The wall mounted air outlet is located diagonally opposite at low level. The chamber was operated at an air flow rate of 0.027 m^3^s^-1^ equivalent to three air-changes-per-hour (ACH). Previous studies have shown the chamber to have good air mixing^40^. The release location of the aerosolized *Staphylococcus aureus* was at a height of 168 cm from the ground, 50 cm from the air inlet and 64 cm from the adjacent wall (Fig. 1). The sample collection point was at a height of 50 cm, positioned 20 cm from the air outlet and 64 cm from the adjacent wall. Prior studies have indicated that this location is representative of the average concentration within the chamber. Care was taken to ensure the bacteria release point and sample point were not located directly under a Far-UVC source (Fig. 1). The chamber was operated at a temperature of 28 °C ± 1 °C and relative humidity 50% ± 2%. There was no air mixing fan within the chamber, although the mechanical ventilation provided a well-mixed air flow^40^. As a biocontainment facility, experiments were conducted with the chamber sealed and nebulisation, aerosol sampling and operation of the Far-UVC devices were carried out remotely.

### Choice of aerosolized pathogen

In practice, the bioaerosol chamber could not be used with aerosolized level-3 pathogens such as SARS-CoV-2. In order to choose a usable aerosolized pathogen which would be a reasonable but conservative model for airborne human coronavirus, we undertook some preliminary studies using the Columbia University laboratory-based aerosolized pathogen UV irradiation system, as described by Welch et al.^28^. This system consists of a source of aerosolized pathogens which flows past a UV irradiation chamber consisting of a far-UVC source and a far-UVC-transparent window; different far-UVC doses are obtained by varying the intensity of the far-UVC exposure and the velocity of the pathogen. The airborne *S. aureus* was collected after irradiation on gelatine filters (Sartorius, Germany) dissolved in 5 ml PBS and assayed for inactivationwith the colony forming unit (CFU) assay on tryptone soy agar (TSA) plates.

Figure 4 shows the in-chamber results for aerosolized *S. aureus* compared with earlier published results for aerosolized human coronaviruses 229E and OC43 and H1N1 influenza virus^27,28^. We concluded from these preliminary studies that, despite the differences between bacteria and viruses, the use of aerosolized *Staphylococcus aureus* in the current room-chamber studies represents a reasonable but conservative model for Far-UVC inactivation of human coronavirus. *S. aureus* has been used by the research team in a number of previous room-scale chamber studies and is shown to be a reliable test organism which can be nebulised into the room and sampled from the space consistently allowing good comparison between experiments^39,40^. *S. aureus* is also a pathogen of interest in itself as it is representative of Methicillin-resistant Staphylococcus aureus (MRSA), which is important in hospital infections and is regularly used as a reference for cleanliness. Assessment of technology for pathogen inactivation beyond the current pandemic was also thought to be vital.

### Far-UVC lamps

Five commercially available Krypton Chloride excimer Far-UVC lamps, filtered to reduce ultraviolet emissions at wavelengths longer than 230 nm, were positioned in a quincunx pattern (the pattern of the five spots on a six-sided dice) within the chamber at a height of 2.12 m, with emission directed towards the ground. The lamps operated continuously and were modified to include a diffusing material which broadened the Far-UVC distribution, maximising the irradiated volume. To adjust the intensity of the lamp emission, metal-mesh attenuation filters were custom made by the Medical Physics and Clinical Engineering department at Ninewells Hospital, Dundee. These filters provided nominal emissions of 10% and 1% of the full Far-UVC intensity. The attenuation filters were placed between the lamp and the diffusing material.

The irradiance (E) field in the chamber was measured in the horizontal plane with a calibrated UVC radiometer (UV-3727-5 detector with X1-5 optometer calibrated 29^th^ April 2021, Gigahertz-Optik, Germany). Measurements were made at two heights (z) from the ground, 1.7 m and 1 m, in 0.5 m intervals throughout the chamber. Exposure is defined in Eq. (1) as the radiant exposure H at height z, such that

1 $${H}_{z}=\int {E}_{iz}d{t}_{i}$$

where E_iz_ is the irradiance at height z and position i in the chamber multiplied by the time spent at position iz, integrated for all positions. The *peak* exposure dose assumes a t of 8 h (28,800 s) at position i where the irradiance measurement is highest for a given z (i.e. directly under the lamp). The *average* exposure dose is defined as the 8-h radiant exposure for the average measured irradiance at the given height. We have made a deliberate decision not to name the Far-UVC device used in this research as these experiments are an investigation of the principle of Far-UVC and not an endorsement of a particular device.

### Experiment procedures

#### Preparation of suspension fluid and bioaerosol generation

The generation of aerosols was performed under a controlled environment in which both temperature (28 °C ± 1 °C) and relative humidity (50% ± 2%) are taken into account. The generation of aerosols was performed using a Collison 6-jet nebuliser (BGI, USA) that operates at a flow rate of 12 L/min and is located externally to the chamber; the aerosol enters the chamber through a tube. The nebuliser generates aerosols in the range of 0.3 µm to 5 µm in diameter, with a mass mean aerodynamic diameter 2.5 µm (geometric standard deviation 1.8). The suspension fluids (100 ml) inside the nebuliser vessel were roughly 1.35 × 10^6^ cfu ml^-1^ concentration of *Staphylococcus aureus* (ATCC 6538) that was dispensed in sterilised distilled water. Preliminary investigation of pathogen suspension in other materials (i.e. 1% Foetal Bovine Serum) demonstrated no significant effect on results.

#### Bioaerosol sampling

The sampling process was performed using an Anderson 6-stage impactor (Anderson INC.) at a flow rate of 28L min^-1^. Samples were taken externally to the chamber, with the sample taken through a tube. The tryptone soy agar (TSA) plates inside the stages 5 and 6 of the Anderson impactor were prepared using 40 g of TSA (Oxoid, UK) for each 1L of distilled water. Approximately 90% of the S. aureus aerosols were collected at stage 6 (aerosol diameters 0.65 µm—1.1 µm) with the remaining 10% collected at stage 5 (aerosol diameters 1.1 µm–2.1 µm). In previous experiments in the same environment, and at the same concentration of S. aureus, the aerosols collected at stages 1–4 (2.1, 3.3, 4.7 and 7 µm) represented less than 1% of total collections. After sampling, the agar plates were incubated at a temperature of 37 °C for 24 h. The Gallenkamp colony counter was then used to count the number of colonies on each plate. Finally, positive-hole correction tables were used to correct the results and the sampler flow rate was used to determine the concentration in air in terms of colony forming units per m^3^^50^.

#### The experiment

The airborne *Staphylococcus aureus* was allowed to establish a steady state within the chamber over a period of 60 min. This steady state is similar to having an infected individual in the corner of the room emitting aerosolised pathogen into the room. Then, ten air samples of four-minute duration were taken every five minutes at the collection point (Fig. 2), with the other minute being used to prepare the next sample. The Far-UVC lamps were then switched on and the sampling was repeated in the same manner. An average of the first ten air samples was used to determine the concentration of *Staphylococcus aureus* (cfu m^-3^) present in the chamber prior to switching on of the Far-UVC lamps. The concentration (cfu m^-3^) of each subsequent air sample was then plotted as a percentage of the average initial steady state concentration.

#### Analysis

Concentrations of *Staphylococcus aureus* were normalised by comparing to the mean concentration of all samples prior to switching on the Far-UVC devices to enable comparison within and between experiments. Steady state concentrations with the lamps switched on were determined from the six measurements taken between 20 and 50 min when the decay period after switch on had ended. The equivalent air change rate due to the Far-UVC was calculated from Eq. (2), the steady state concentrations before (*C*) and after (*C*_*uv*_) the lamps were switched on.

2 $$\frac{C}{{C}_{uv}}=\frac{N+{N}_{uv}}{N}$$

Here *N* is the ventilation rate of the room (ACH) and *N*_*uv*_ is the equivalent air change rate (eACH) due to the Far-UVC.

#### Statistical analysis

Unpaired t-tests were used to compare viable pathogen before and 20 min after Far-UVC lamps were switched on. Statistical analyses were carried out using GraphPad Prism (Prism 9, GraphPad Software, USA). In all cases, statistical significance is represented by: ns = *p* > 0.05, * = *p* ≤ 0.05, ** = *p* ≤ 0.01, *** = *p* ≤ 0.001, and **** = *p* ≤ 0.0001.

## Supplementary Information

Supplementary Information.

## Data Availability

All data generated or analysed during this study are included in this published article (and its Supplementary Information files).
