# Supplementary material for: Far-UVC (222 nm) efficiently inactivates an airborne pathogen in a room-sized chamber
Source: Sci Rep. 2022 Mar 23;12:4373. doi: 10.1038/s41598-022-08462-z (PMC8943125; doi:10.1038/s41598-022-08462-z)
Supplement: Supplementary file 1 — Supplementary Information. [file 41598_2022_8462_MOESM1_ESM.pdf]

Supplementary Information

Far-UVC efficiently inactivates an airborne pathogen in a room-sized chamber

Eadie, Ewan.[1]*, Hiwar, Waseem.[2], Fletcher, Louise.[2], Tidswell, Emma.[2], O'Mahoney, Paul.[1,3], Buonanno, Manuela.[4], Welch, David.[4], Adamson, Catherine S.[5], Brenner, David J.[4], Noakes, Catherine.[2], Wood, Kenneth.[6]

[1] NHS Tayside, Photobiology Unit, Ninewells Hospital and Medical School, Dundee, DD1 9SY
[2] School of Civil Engineering, University of Leeds, Leeds, LS2 9JT
[3] University of Dundee, School of Medicine Ninewells Hospital and Medical School, Dundee, DD1 9SY
[4] Center for Radiological Research, Columbia University Medical Center, New York, NY.
[5] School of Biology, Biomedical Sciences Research Complex, University of St Andrews, North Haugh, St Andrews, KY16 9ST
[6] SUPA, School of Physics & Astronomy, University of St Andrews, North Haugh, St Andrews, KY16 9SS

Corresponding author: ewan.eadie@nhs.scot

Supplementary S1

Table S1: Results of unpaired t-test on viable pathogen (cfu m^{-3}) before Far-UVC Lamp switch on (SO) and from 25 – 50 minutes after lamp SO.

		Unpaired t-test						
		Before SO		After SO		df	t(df)	p
		Mean (cfu m^{-3})	SD (cfu m^{-3})	Mean (cfu m^{-3})	SD (cfu m^{-3})			
High	1 Lamp	3491	736.4	220.8	34.10	14	10.72	<0.0001
	5 Lamps	23761	2239	381.7	162.1	14	25.18	<0.0001
Medium	1 Lamp	3741	457.1	1277	150.4	9	12.53	<0.0001
	5 Lamps	3927	280.4	314	37.05	14	30.96	<0.0001
Low	1 Lamp	8260	1334	7207	316.4	12	1.878	0.0849
	5 Lamps	10684	2466	7621	358.8	12	2.990	0.0113

Supplementary S2

Experiment Data

	cfu m^{-3}						
	High		Medium		Low		Repeat High
Time from lamp switch on (mins)	1 Lamp	5 Lamp	1 Lamp	5 Lamp	1 Lamp	5 Lamp	5 Lamp
-45	4160.7	2035.7		3660.7			25750.0
-40	3321.4	2937.5		3830.4			21142.9
-35	3357.1	2250.0		3607.1	8401.8	7864.3	21642.9
-30	2383.9	2142.9		4196.4	11383.9	10297.6	26178.6
-25	2178.6	2339.3		3910.7	7803.6	12696.4	25107.1
-20	3830.4	2375.0	3321.4	3580.4	7840.1	8616.1	24714.3
-15	4200.7	1910.7	3848.2	3892.9	8419.6	14125.0	24107.1
-10	4366.1	2098.2	3758.9	4419.6	7348.2	13705.4	26178.6
-5	3705.4	2901.8	4437.5	3946.4	7232.1	9241.1	22821.4
0	3401.8	2303.6	3339.3	4223.2	7652.0	8928.6	19964.3
5	535.7	26.8	3196.4	2794.6	7794.6	8785.7	
10	276.8	17.9	2714.3	767.9	7732.1	7303.6	
15	285.7	8.9	2285.7	339.3	7919.6	7714.3	
20	178.6	9.0	1741.1	339.3	8214.3	7517.9	500.0
25	214.3	0.0	1526.8	250.0	7250.0	7723.2	678.6
30	187.5	0.0	1330.4	312.5	7776.8	6973.2	205.4
35	178.6	0.0	1285.7	339.3	7205.4	7821.4	383.9
40	226.2	8.9	1267.9	348.2	7000.0	8017.9	333.9
45	258.9	0.0	1151.8	294.6	7160.7	7508.9	401.8
50	258.9	0.0	1098.2	339.3	6848.2	7678.6	285.7
Average lamps off	3490.6	2329.5	3741.1	3926.8	8260.2	10684.3	23760.7
Average lamps on (after 20 mins)	220.7	1.5	1276.8	314.0	7206.8	7620.5	381.5
% reduction	93.7	99.9	65.9	92.0	12.8	28.7	98.4
St Dev	34.3	3.6	150.4	37.2	316.3	358.8	162.0
St Dev (%)	1.0	0.2	4.0	0.9	3.8	3.4	0.7
2 St Dev	2.0	0.3	8.0	1.9	7.7	6.7	1.4
Range (+1 St Dev)	94.7	100.1	69.9	93.0	16.6	32.0	99.1
Range (-1 St Dev)	92.7	99.8	61.9	91.1	8.9	25.3	97.7
eACH (Mean)	44	4,693	6	35	0	1	184
eACH (Mean + 1 SD)	53	N/A	7	40	1	1	322
eACH (Mean - 1 SD)	38	1,358	5	31	0	1	128
